# Galectin-3 as a potential prognostic biomarker of severe COVID-19 in SARS-CoV-2 infected patients

**DOI:** 10.1038/s41598-022-05968-4

**Published:** 2022-02-03

**Authors:** Eduardo Cervantes-Alvarez, Nathaly Limon-de la Rosa, Moises Salgado-de la Mora, Paola Valdez-Sandoval, Mildred Palacios-Jimenez, Fatima Rodriguez-Alvarez, Brenda I. Vera-Maldonado, Eduardo Aguirre-Aguilar, Juan Manuel Escobar-Valderrama, Jorge Alanis-Mendizabal, Osvely Méndez-Guerrero, Farid Tejeda-Dominguez, Jiram Torres-Ruíz, Diana Gómez-Martín, Kathryn L. Colborn, David Kershenobich, Christene A. Huang, Nalu Navarro-Alvarez

**Affiliations:** 1 Department of Gastroenterology, Instituto Nacional de Ciencias Médicas y Nutrición Salvador Zubirán, Vasco de Quiroga #15, Tlalpan, Mexico City, Mexico; 2 Department of Internal Medicine, Instituto Nacional de Ciencias Médicas y Nutrición Salvador Zubirán, Mexico City, Mexico; 3 Department of Immunology and Rheumatology, Instituto Nacional de Ciencias Médicas y Nutrición Salvador Zubirán, Mexico City, Mexico; 4 Universidad Panamericana School of Medicine, Campus México, Mexico City, Mexico; 5 PECEM, Facultad de Medicina, Universidad Nacional Autónoma de México, Mexico City, Mexico; 6 Universidad Veracruzana, Veracruz, Mexico; 7 Department of Surgery, University of Colorado Anschutz Medical Campus, Denver, CO USA

**Keywords:** Predictive markers, Viral infection

## Abstract

Severe COVID-19 is associated with a systemic hyperinflammatory response leading to acute respiratory distress syndrome (ARDS), multi-organ failure, and death. Galectin-3 is a ß-galactoside binding lectin known to drive neutrophil infiltration and the release of pro-inflammatory cytokines contributing to airway inflammation. Thus, we aimed to investigate the potential of galectin-3 as a biomarker of severe COVID-19 outcomes. We prospectively included 156 patients with RT-PCR confirmed COVID-19. A severe outcome was defined as the requirement of invasive mechanical ventilation (IMV) and/or in-hospital death. A non-severe outcome was defined as discharge without IMV requirement. We used receiver operating characteristic (ROC) and multivariable logistic regression analysis to determine the prognostic ability of serum galectin-3 for a severe outcome. Galectin-3 levels discriminated well between severe and non-severe outcomes and correlated with markers of COVID-19 severity, (CRP, NLR, D-dimer, and neutrophil count). Using a forward-stepwise logistic regression analysis we identified galectin-3 [odds ratio (OR) 3.68 (95% CI 1.47–9.20), *p* < 0.01] to be an independent predictor of severe outcome. Furthermore, galectin-3 in combination with CRP, albumin and CT pulmonary affection > 50%, had significantly improved ability to predict severe outcomes [AUC 0.85 (95% CI 0.79–0.91, *p* < 0.0001)]. Based on the evidence presented here, we recommend clinicians measure galectin-3 levels upon admission to facilitate allocation of appropriate resources in a timely manner to COVID-19 patients at highest risk of severe outcome.

## Introduction

Coronavirus disease 2019 (COVID-19) caused by severe acute respiratory syndrome coronavirus 2 (SARS-CoV-2) infection has afflicted tens of millions of people in a worldwide pandemic, straining health care systems across the world^1,2^. Prognostic biomarkers that can identify high-risk patients are needed to improve clinical management and allow appropriate allocation of healthcare resources. Moreover, the lack of current curative therapies emphasizes the need to get a better understanding of the pathophysiological process behind SARS-CoV-2 infection and its long-term consequences for the development of targeted therapeutic strategies.

Severe COVID-19 is associated with a systemic hyperinflammatory response characterized by high levels of circulating cytokines and chemokines^3^ and substantial lung infiltration of innate immune cells^4^ that can lead to acute respiratory distress syndrome (ARDS), multi-organ failure and death^5,6^. Among the inflammatory cytokines are those associated with the activation of monocyte/macrophages such as Interleukin 6 (IL-6), Tumor necrosis factor (TNF), and the CC-chemokine ligand 2 (CCL2)^3,6,7^.

Studies have shown that those inflammatory cytokines contribute to the recruitment of additional inflammatory cells that not only aggravate the lung damage, but also lead to pulmonary fibrosis^8,9^. Subsets of M2 macrophages expressing profibrogenic genes have been found in the bronchoalveolar lavage of COVID-19 patients^4^, reflecting that the pathological process of SARS-CoV-2 infection not only involves an acute inflammatory response in the lungs, but is also associated with fibrotic complications^10^.

Galectin-3 is a 29–35 kDa ß-galactoside binding lectin^11^ known to enhance the effects of viral infection by promoting host inflammatory responses^12,13^ and the release of several cytokines including IL-6 and TNF-α^14^, which are some of the major cytokines present in severe COVID-19 patients^3^. High levels of galectin-3 have been shown to drive neutrophil infiltration contributing to acute airway inflammation^15–17^, and are associated with disease severity and mortality in ARDS patients^18^. Galectin-3 is increasingly recognized as a potentially important diagnostic or prognostic biomarker for a variety of inflammatory and fibrotic diseases^19–21^ and has been found to be elevated in patients with idiopathic pulmonary fibrosis^22,23^ and more recently in COVID-19 patients^24,25^. Inflammation and fibrosis are key contributing mechanisms to the progression of severe COVID-19 and the development of its long-term consequences^3,10,26^ .

Given the known proinflammatory and profibrotic roles of galectin-3, the aim of this study was to analyze the prognostic value of serum galectin-3 upon hospital admission to predict patients at high-risk of progressing to a severe COVID-19 outcome resulting in invasive mechanical ventilation (IMV) and/or death.

## Methods

### Study design and population

This single-center, prospective observational study was performed in COVID-19 patients admitted to the Instituto Nacional de Ciencias Médicas y Nutrición Salvador Zubirán (INCMNSZ), one of the largest designated institutions in Mexico for the hospitalization of patients with COVID-19 between April and October 2020. The inclusion criteria were patients ≥ 18 years with laboratory confirmed COVID-19 by real-time reverse transcriptase-polymerase chain reaction (RT-PCR). We excluded pregnant women.

### Primary outcome definition

Patients who required IMV and/or died during hospitalization were categorized as having a *severe outcome*. Patients who recovered and were discharged without requiring IMV were categorized as having a *non-severe outcome*.

### Data collection

Clinical and laboratory data were extracted from the electronic medical records including: Demographics (age, gender, and comorbidities), clinical (days of hospital stay), radiological (chest CT findings), laboratory and patient outcome data (need for IMV and/or death). Laboratory data included, complete blood count, triglycerides, albumin, AST, International Normalized Ratio (INR), thrombo-inflammatory markers (D-dimer, fibrinogen and ferritin) and C-reactive protein (CRP). All information was recorded in a specific database. Data was independently reviewed by 2 investigators to verify the correct collection of the data.

### Sample collection and Galectin-3 levels measurement

Blood samples were collected upon hospital admission from all COVID-19 patients fulfilling inclusion criteria. Samples were centrifuged at 3000 rpm for 10 min, and serum was aliquoted and stored at − 70 °C until further analysis. Galectin-3 was measured in the serum samples using a commercial enzyme-linked immunosorbent assay (ELISA) Kit (Invitrogen, #BMS 279-4, Carlsbad, CA, USA), according to the manufacturing instructions. The detection limit of this kit is 0.29 ng/mL and a mean recovery of 100% after spike recovery and linearity of dilution assessments is reported. All samples were evaluated in duplicate. The inter-assay coefficient of variation was 8.52% and the intra-assay 5.34%. Galectin-3 levels in COVID-19 patients were compared against those of age-matched healthy control subjects analyzed before the pandemic, between 2018 and 2019.

### Statistical analysis

Data are expressed as frequencies for categorical variables and as mean with standard deviation (SD) or median with interquartile range (IQR) for continuous variables according to their normality as assessed by the Kolmogorov–Smirnov test. Student’s t-tests or Mann–Whitney U tests were used for univariate statistical comparisons, while correlation analyses were performed with Spearman's correlation coefficient for pairs of continuous variables. To determine the prognostic ability of galectin-3 and inflammatory markers for the primary outcome, receiver operating characteristic (ROC) curves were plotted, and cut-point values were chosen as those with the highest Youden’s *J* statistic. Comparisons between AUCs obtained were performed with DeLong's test for correlated ROC curves. Independent predictors of the primary outcome were determined after performing a multivariable logistic regression analysis with the forward-stepwise selection method. Analyzed variables were those with a p value < 0.20 after univariate analyses. Only those variables chosen by the stepwise procedure were reported in Table 3. Goodness of the fit was assessed with the Hosmer–Lemeshow test. The combined power of the identified independent predictors was evaluated with a ROC curve using the model selected by the stepwise logistic regression procedure. Statistical analyses were performed with SPSS (version 24.0, SPSS Inc., Chicago, IL, USA) and GraphPad Prism (version 8.00, GraphPad Software, La Jolla, CA, USA). A selected alpha level of 0.05 indicated statistical significance.

### Ethics approval and consent to participate

This study was approved by INCMNSZ’s Research Ethics Committee (No. GAS-3385-20-20-1) and complied with the provisions of the Declaration of Helsinki. Informed written consent was obtained from all patients prior to blood sample collection.

## Results

### Demographic, clinical and laboratory characteristics

A total of 156 patients with RT-PCR-confirmed SARS-CoV-2 infection and CT findings were enrolled in the study. The mean age in the overall population was 53.24 ± 13.22 years, of which 107 (68.6%) were male and 49 (31.4%) females. Based on our primary outcome definition, 54 (34.6%) patients progressed to a *severe outcome* and 102 (65.4%) to a *non-severe outcome* (Supplementary Fig. S1).

There were no differences in age, gender, or body mass index (BMI) between patients with severe and non-severe outcome. The principal comorbidities among our cohort were obesity (44.2%), hypertension (30.1%) and diabetes (21.2%), all of which had been diagnosed prior to hospital admission.

Patients with a severe outcome had a significantly longer hospital stay compared to non-severe outcome patients (16 [6–26] vs. 7 [5–9], *p* < 0.0001). From the 54 patients with severe outcome, 47 (87%) had a critical disease with > 50% of lung damage, and only 7 (13%) presented a moderate disease with < 50% of lung damage. Whereas from the 102 patients with non-severe outcome, 47 (46.1%) had critical disease and 55 (53.9%) moderate disease. (Table 1).

**Table 1:** Demographic, clinical and laboratory characteristics.

	Total (n = 156)	Severe outcome (n = 54)	Non-severe outcome (n = 102)	*p* value
Age	53.24 ± 13.22	54.63 ± 11.52	50.97 ± 13.92	0.10
Gender, male	107 (68.6%)	42 (39.3%)*	65 (60.7%)*	0.07
Gender, female	49 (31.4%)	12 (24.5%)*	37 (75.5%)*	
BMI	29.42 (26.74–33.78)	29.35 (26.79–32.89)	29.42 (26.80–33.40)	0.77
Normal	21 (13.5%)	4 (19.0%)*	17 (81.0%)*	0.14
Overweight	66 (42.3%)	25 (37.9%)*	41 (62.1%)*	0.46
Obesity	69 (44.2%)	25 (36.2%)*	44 (63.8%)*	0.71
Hypertension	47 (30.1%)	17 (36.2%)*	30 (63.8%)*	0.96
Diabetes	33 (21.2%)	15 (45.5%)*	18 (54.5%)*	0.23
Alcohol consumption	60 (38.5%)	24 (40.0%)*	36 (60.0%)*	0.28
Days of hospital stay	7 (5–14)	16.5 (6–26)	7 (5–9)	< **0.0001**
IMV	51 (32.7%)	51 (94.4%)	0 (0.0%)	< **0.0001**
Mortality	21 (13.5%)	21(38.9%)	0 (0.0%)	< **0.0001**
**CT pulmonary affection**				
> 50%, critical disease	94 (60.3%)	47 (87.0%)	47 (46.1%)	< **0.0001**
< 50%, moderate disease	62 (39.7%)	7 (13.0%)	55 (53.9%)	
**Laboratory tests**				
Galectin-3 (ng/ml)	28.77 (17.52–42.04)	41.17 (29.71–52.25)	23.76 (15.78–33.97)	< **0.0001**
Neutrophil count (× 10^3^/µl)	6.83 (4.13–10.61)	9.4 (5.98–14.28)	6 (3.54–9)	< **0.0001**
Lymphocyte count (× 10^3^/µl)	0.77 (0.53–1.05)	0.7 (0.48–0.98)	0.84 (0.57–1.09)	0.09
NLR	8.43 (5.29–17.68)	14.22 (7.94–23.32)	6.97 (4.09–12.89)	< **0.0001**
Fibrinogen	645 (482–772)	682 (592.5–848.75)	616.5 (455.5–738)	< **0.01**
Ferritin (ng/ml)	500.47 (292.25–1020.5)	722.7 (339.4–1167)	376.4 (227.3–851.6)	< **0.01**
D-Dimer (ng/ml)	698.5 (459.5–1178.5)	1176 (528.8–2510)	604.5 (400.8–928.8 )	< **0.0001**
CRP (mg/dl)	14 (6.13–23.01)	20.96 (14.42–29.17)	9.38 (4.48–19.1)	< **0.0001**
INR	1.1 (1–1.2)	1 (1.1–1.2)	1.1 (1–1.2)	**0.03**
Platelets (K/μl)	224.25 (191–288)	220.7 (185.5–300)	234 (193–290)	0.66
Albumin (g/dl)	3.7 ± 0.54	3.44 ± 0.47	3.89 ± 0.49	< **0.0001**
AST (U/L)	36.25 (26.8–59.9)	43.26 (33–65)	31.62 (23.1–56.7)	< **0.001**
Triglycerides (mg/dL)	152 (115.5–199.5)	175 (127.8–224.8)	137.5 (112–193.3)	**0.011**

Data are reported as median (IQR), mean (± SD) and n (%).Comparisons were performed with Pearson’s Chi-squared test or Fisher’s exact test and either Student’s t-test or Mann–Whitney U test. Bold values represent *p* < 0.05.

*BMI* Body mass index.

Laboratory characteristics including complete blood count, inflammatory and thrombo-inflammatory markers, AST and coagulation tests for both groups are depicted in Table 1.

### Galectin-3 serum levels are higher in COVID-19 patients with a severe outcome

**Figure 1:**
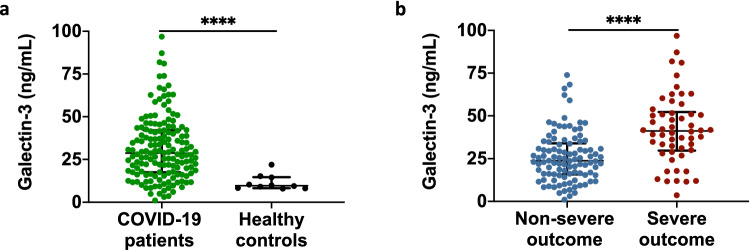
Galectin-3 serum levels in COVID-19 patients. (**a**) Galectin-3 circulating levels upon hospital admission of COVID-19 patients (n = 156) and age-matched healthy pre-pandemic controls (n = 10). (**b**) Severe outcomes in COVID-19 patients were associated with elevated levels of galectin-3. Data in (**a**, **b**) are shown as median with IQR. ****p < 0.0001; two-tailed Mann–Whitney U test or two-tailed t-test. Samples were assessed in duplicate in ELISA assays.

To explore the possible role of galectin-3 as a biomarker of severity, circulating levels were measured in sera from COVID-19 patients using a commercial ELISA kit. We found that COVID-19 patients upon hospital admission had significantly elevated circulating levels of galectin-3 when compared to age-matched pre-pandemic healthy subjects (28.77 ng/mL [17.52–42.04] vs. 9.65 ng/mL [8.27–14.71], *p* < 0.0001) (Fig. 1a). Patients who developed a severe outcome, including IMV and/or in-hospital death, had significantly higher galectin-3 levels than those with a non-severe outcome (41.17 ng/mL [29.71–52.25] vs. 23.76 ng/mL [15.78–33.97], *p* < 0.0001) (Fig. 1b).

### Galectin-3 correlates with other inflammatory biomarkers

**Figure 2:**
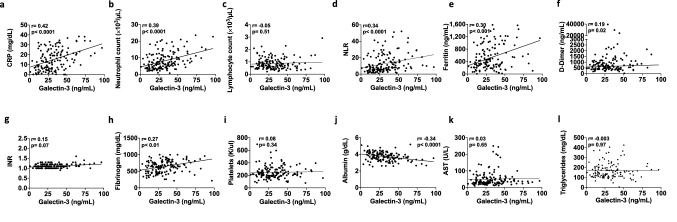
Galectin-3 correlates with inflammatory markers in COVID-19 patients. Spearman correlations show significant associations between galectin-3 and commonly measured inflammatory markers in SARS-CoV-2 infected patients. (**a**) CRP, (**b**) Neutrophil count, (**c**) Lymphocyte count, (**d**) NLR, (**e**) Ferritin, (**f**) D-dimer, (**g**) INR, (**h**) Fibrinogen, (**i**) Platelets, (**j**) Albumin, (**k**) AST and (**l**) Triglycerides.

Given that elevated galectin-3 levels were found in patients who progressed to a severe outcome, and considering its participation in the inflammatory response, correlations with inflammatory parameters previously studied in COVID-19 using the spearman correlation coefficient, in accordance with the non-normal distribution of the data were carried out. We found that galectin-3 correlates positively with CRP (r = 0.42, *p* < 0.0001), neutrophil count (r = 0.39, *p* < 0.0001), NLR (r = 0.34, *p* < 0.0001), ferritin (r = 0.30, *p* < 0.001), D-dimer (r = 0.19, *p* = 0.02) and fibrinogen (r = 0.27, *p* < 0.01), whereas a negative correlation was found with albumin (r = 0.35, *p* < 0.0001) (Fig. 2a–l).

### Galectin-3 in combination with CRP, albumin and CT pulmonary affection accurately predicts severity in COVID-19 patients

**Figure 3:**
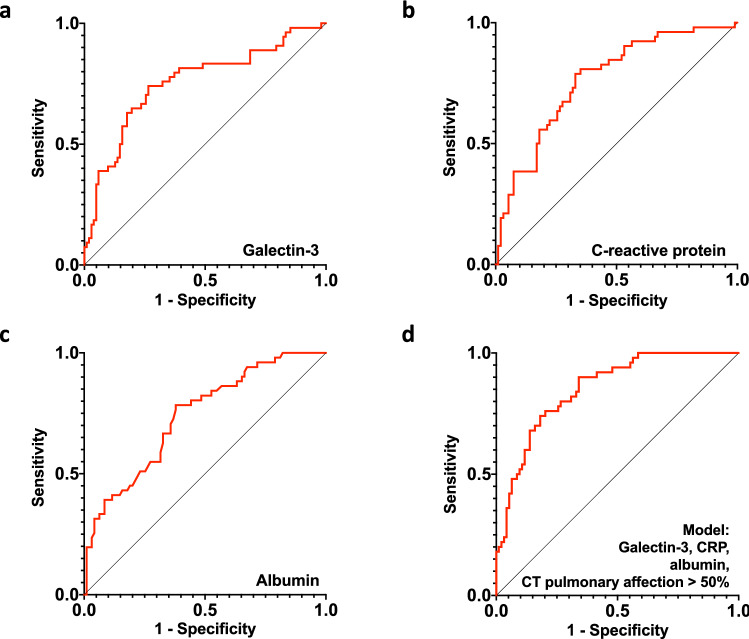
Galectin-3, CRP, albumin and CT pulmonary affection > 50% as independent predictors of severe outcome in COVID-19 patients. Receiver-operating characteristic curves (ROCs) of the independent predictors for the classification of binary outcomes (severe/non-severe) using (**a**) galectin-3, (**b**) CRP, (**c**) albumin and (**d**) the combined predicted probabilities of galectin-3, CRP albumin and CT pulmonary affection > 50%.

To assess the discriminative ability of galectin-3 as a predictor of severe outcome, ROC curves were plotted. Galectin-3 discriminates well between those with severe and non-severe outcome, with an AUC of 0.75 (95% CI 0.67–0.84, *p* < 0.0001), and a cut-point of 30.99 ng/mL (74.07% sensitivity, 73.53% specificity) (Fig. 3a). Other inflammatory and thromboinflammatory parameters studied in COVID-19 could also discriminate patients with severe outcome, except for lymphocyte count and platelets (Table 2).

**Table 2:** Discrimination power of other inflammatory and thromboinflammatory markers for severe outcome and comparison with galectin-3.

Biomarker	AUC	95% CI	*p* value	*p* value (comparison with galectin-3 AUC)
Galectin-3	0.75	(0.67–0.84)	< **0.0001**	–
CRP	0.76	(0.68–0.85)	< **0.0001**	0.70
Albumin	0.73	(0.65–0.82)	< **0.0001**	0.93
NLR	0.71	(0.62–0.79)	< **0.0001**	0.52
Neutrophil count	0.70	(0.61–0.78)	< **0.0001**	0.43
Ferritin	0.66	(0.57–0.76)	< **0.01**	0.19
Lymphocyte count	0.58	(0.49–0.68)	0.09	0.02
Platelets	0.48	(0.38–0.58)	0.66	< 0.01
D-Dimer	0.69	(0.60–0.79)	< **0.001**	0.49
Fibrinogen	0.66	(0.57–0.75)	< **0.01**	0.16
INR	0.61	(0.51–0.71)	**0.04**	0.05
Triglycerides	0.64	(0.54–0.74)	**0.01**	0.12
AST	0.68	(0.59–0.76)	< **0.001**	0.27

ROC curves were performed to determine the discrimination power of each biomarker for severe outcome. The AUC obtained was compared against that of galectin-3 using DeLong’s test for correlated ROC curves. Bold values represent *p* < 0.05.

Based on the above, we performed a forward-stepwise logistic multivariate regression analysis to identify independent demographic and laboratory parameters that predict and strongly correlated with severe outcome, and thus with disease progression (i.e., IMV and/or death). A smoothing spline of galectin-3 showed a non-linear relationship with severe outcome; therefore, we used the Youden’s *J* statistic to determine the ideal binary cut-point of galectin-3 for classifying severe outcomes (Supplementary Fig. S2a). Variables assessed in univariate analyses included age, gender, comorbidities and inflammatory parameters (Table 3). The final model selected via the stepwise procedure included galectin-3 (odds ratio [OR] 3.68 [95% CI 1.47–9.20], *p* < 0.01), critical disease (CT pulmonary affection > 50%) (4.04 [95% CI 1.17–13.97], *p* = 0.03), CRP (OR 1.05 [95% CI 1.00–1.11], *p* = 0.04), and low albumin levels upon admission (OR 0.35 [95% CI 0.13–0.89], *p* = 0.03) when regressed on severe outcome (IMV and/or death; Hosmer–Lemeshow goodness of fit test *p* = 0.74; Table 3). Of note, CRP and albumin were entered as continuous variables according to their smoothing splines which showed linear relationships with severe outcome (Supplementary Fig. S2b,c). The equation fitted by this logistic regression model was the following, where $p = P\left( {Y = 1} \right)$: $\log \frac{p}{1 - p}$ = 0.649 + [1.302 * binary galectin-3] + [1.396 * CT pulmonary affection] + [0.052 * CRP] − [1.062 * albumin], where galectin-3 is coded as binary (less than 30.99 ng/mL with 0 and above 30.99 ng/mL with 1) as well as CT pulmonary affection (< 50% or moderate disease with 0 and > 50% or critical disease with 1) and CRP and albumin as continuous variables. The obtained values were transformed into predicted probabilities with the formula exp ($\log \frac{p}{1 - p}$)/(1 + exp ($\log \frac{p}{1 - p}$)).

**Table 3:** Univariate and multivariable logistic regression analyses for severe outcome.

Variable	Univariate			Multivariable			*p* value (individual AUC vs. model AUC)
	Odds ratio	95% CI	*p* value	Odds ratio	95% CI	*p* value	
Galectin-3 (binary)	7.94	(3.75–16.82)	< **0.0001**	3.68	(1.47–9.20)	< **0.01**	< **0.001**
CRP	1.11	(1.07–1.16)	< **0.0001**	1.05	(1.00–1.11)	**0.04**	**0.03**
Albumin	0.14	(0.06–0.33)	< **0.0001**	0.35	(0.13–0.89)	**0.03**	**0.03**
Critical disease (CT pulmonary affection > 50%)	7.86	(3.25–19.03)	< **0.0001**	4.04	(1.17–13.97)	**0.03**	< **0.0001**
Gender (male)	1.99	(0.93–4.25)	**0.08**				
Age	1.02	(1.00–1.05)	**0.10**				
Diabetes	1.62	(0.74–3.57)	0.23				
Hypertension	0.98	(0.48–2.01)	0.96				
Obesity (BMI ≥ 30)	1.14	(0.59–2.21)	0.71				
NLR	1.05	(1.02–1.09)	< **0.01**				
Neutrophil count	1.15	(1.07–1.23)	< **0.001**				
Ferritin	1.00	(1.07–1.23)	< **0.01**				
Lymphocyte count	0.63	(0.28–1.42)	0.27				
Platelets	1.00	(1.00–1.00)	0.89				
D-Dimer	1.00	(1.00–1.00)	**0.03**				
Fibrinogen	1.00	(1.00–1.01)	< **0.01**				
INR	2.06	(1.01–4.24)	**0.05**				
Triglycerides	1.01	(1.00–1.01)	**0.02**				
AST	1.01	(1.00–1.02)	**0.03**				

Galectin-3 was analyzed as a binary variable according to its non-linear relationship with severe outcomes (> 30.99 ng/mL = 1, < 30.99 ng/mL = 0). Only variables with a *p* value < 0.20 after univariate analyses were further evaluated in multivariable analyses. The AUC of the final model was compared against that of each independent predictor with DeLong’s test for correlated ROC curves. Bold values represent *p* < 0.05.

We also explored the binary cut-points of the other two biomarkers obtained from the model, (CRP and albumin), to classify severe outcomes (Fig. 3b,c, respectively). CRP had an AUC of 0.76 (95% CI 0.68–0.85, *p* < 0.0001) and a cut-point of 14.04 mg/dL (78.85% sensitivity, 67.02% specificity), while albumin had an AUC of 0.73 (95% CI 0.65–0.82, *p* < 0.0001) with a 3.74 g/dL cut-point (78.43% sensitivity, 62.11% specificity). We then assessed if the model proposed by the logistic regression analyses could better predict severe outcome in COVID-19 patients than either predictor on its own. To determine this, the predicted probabilities for this combination of values were computed and plotted in a ROC curve (Fig. 3d). Its AUC showed an enhanced ability to classify severe outcome (0.85 [95% CI 0.79–0.91], *p* < 0.0001) and was significantly higher compared to the individual AUC of each independent predictor (Table 3).

## Discussion

In this prospective cohort of COVID-19 patients, we assessed the classification performance of circulating galectin-3 levels obtained upon hospitalization on the development of a severe outcome, defined as requirement of IMV and/or death. We hypothesized that this molecule could be associated with symptom severity due to its known involvement in the exacerbated inflammatory response, a feature that has been exhibited in COVID-19 patients^3^.

Evidence in the literature has implicated the cytokine release syndrome as the main factor responsible for the high mortality observed in COVID-19 patients^27^. Disease progression is associated with ARDS characterized by diffuse alveolar damage in the lung caused by the severe inflammatory process^28^. ARDS in COVID-19 leads to more severe outcomes than ARDS due to other causes^29^ with a general mortality of 26–61.5% in those admitted to the intensive care unit, and significantly higher in those requiring IMV (65.7% to 94%)^29^.

Galectin-3 has been shown to orchestrate the inflammatory response syndrome activating immune cells and triggering the release of inflammatory cytokines^30,31^.

This study presents for the first time an important connection between galectin-3 and the hyperinflammatory state in COVID-19 patients. Our observations reveal that higher galectin-3 levels upon admission are found in those patients with severe outcome. Values greater than 30.99 ng/mL have a high sensitivity and specificity to predict an adverse clinical course with the possibility of requiring IMV and/or death which might indicate its possible role in the pathophysiology of ARDS reflecting the excessive inflammatory response associated with this syndrome.

While IMV is intended to minimize the progression of lung injury^32^, it has also been demonstrated to induce or aggravate lung damage and in the long-run may contribute to lung fibrosis^4,33^. Chronic pulmonary fibrosis has been observed in recovered COVID-19 patients^10,26^. Galectin-3 is known to play a role in the pathogenesis of pulmonary fibrosis, and clinical trials testing galectin-3 inhibitors are currently underway for the treatment of idiopathic pulmonary fibrosis^34^. Targeting Galectin-3 has been suggested as treatment for COVID-19 not only due to its role in fibrosis and systemic inflammation, but also due to its potential involvement in the virus-host interaction mediated by the N-terminal domain of SARS-CoV-2^35,36^. Given the known sequence and functional similarities between the N-acetylneuraminic acid binding domain on the spike protein of SARS-CoV-2 and human galectin-3^36^, it is possible that by targeting galectin-3, we might also interfere with viral-host interactions, thus potentially decreasing viral load and the resulting inflammatory responses associated with the infection.

Galectin-3 significantly correlated with several inflammatory and thrombo-inflammatory biomarkers, indicating its pathophysiological implication in COVID-19’s inflammatory response. Many of the classic biomarkers used in COVID-19 including CRP, NLR, Ferritin, neutrophil count, D-dimer, among others, had a significant discriminating ability for severe outcome on their own, however after the selection of variables via forward stepwise in the multivariate, many were no longer significant, likely due to collinearity. However, galectin-3 remained an independent predictor of severe outcome even after adjusting for age, gender, comorbidities, and those other inflammatory parameters. CRP, an acute inflammatory biomarker with ability to predict mortality in COVID-19^37,38^, was also identified as an independent predictor of severe outcome in our cohort and had a positive correlation with galectin-3. This novel association between galectin-3 and CRP has not been reported in viral infection, much less in COVID-19 but it suggests the utility of this molecule in detecting the inflammatory state of patients upon hospital arrival. As both CRP and galectin-3 were identified as independent predictors, we sought to identify which one would perform better according to its association with patient outcome. The non-linear relationship of galectin-3, observed in a smoothing spline, demonstrated that higher levels of this lectin were a common characteristic of patients at high-risk of progressing to a severe COVID-19 outcome. We consider that inflammatory markers such as CRP which tend to show a linear relationship with outcome may not be suitable as efficient biomarkers given their inconsistency, which is reflected in the various cut-off values thus far reported. This may partially explain the fact that although numerous inflammatory biomarkers have been widely described as predictors of severity in COVID-19, their measurement and use in clinical practice is lacking. Measuring Galectin-3 may present an advantage given its accuracy and the simplicity of using a binary cut-point to classify patients as having low or high risk for severe outcomes due to its non-linear spline.

We also identified hypoalbuminemia as a common characteristic among critically ill patients which was negatively correlated with galectin-3. Albumin is an important biomarker that reflects the inflammatory state, as its production is decreased due to higher levels of IL-6^39^. Observations carried out by Huang et al. in a large cohort of COVID-19 patients identified the decrease in albumin levels as a significant indicator of progression to a critical stage and death. They associated this pathologic finding with a reduced capacity of synthesis by hepatocytes as mild hepatic injury was evident^40^. Another aspect relevant to consider is that capillary leakage into the interstitial space increases in severe illness such as sepsis, leading to the sequestration of albumin^41^. As galectin-3 was found to reflect the hyperinflammatory state of patients, its predictive ability together with CRP, albumin and CT pulmonary affection > 50% was tested. Results revealed that when used jointly, severe outcomes can be more accurately classified upon hospital admission (AUC = 0.85), thus providing clinicians more resources to efficiently identify patients with higher odds of adverse progression.

There are some limitations to our study. First, since this is a single-center experience, data from different populations and a multicenter analysis will be needed for validation. Second, due to the small sample size, further clinical studies with larger sample sizes are required to confirm these findings before galectin-3 can definitively be recommended in the hospital setting. Despite these limitations, this study demonstrates in a prospective cohort of COVID-19 patients at one of the largest health institutes in Mexico that measurement of galectin-3 levels upon hospital admission could be helpful in predicting disease progression. Finally, the combined use of galectin-3, CRP, albumin and CT pulmonary affection > 50% showed strong predictive ability, and thus could aid to efficiently allocate medical resources before patients develop an adverse outcome.

## Conclusion

We have offered evidence on the prognostic use of galectin-3 in SARS-CoV-2 infected patients which may extend to other critical diseases and propose its combined use with other inflammatory markers to guide the clinical rationale when assessing a hospitalized patient’s risk.

## Supplementary Information

Supplementary Information.

## Data Availability

The datasets used and/or analyzed during the current study are available from the corresponding author on reasonable request. All data generated during this study are included in this published article.

## Abbreviations

ARDS: Acute respiratory distress syndrome
COVID-19: Coronavirus disease 2019
CRP: C-Reactive protein
CT: Computerized tomography
IMV: Invasive mechanical ventilation
SARS-CoV-2: Severe acute respiratory syndrome coronavirus 2
