# Supplementary material for: Galectin-3 as a potential prognostic biomarker of severe COVID-19 in SARS-CoV-2 infected patients
Source: Sci Rep. 2022 Feb 3;12:1856. doi: 10.1038/s41598-022-05968-4 (PMC8813958; doi:10.1038/s41598-022-05968-4)
Supplement: Supplementary file 1 — Supplementary Information. [file 41598_2022_5968_MOESM1_ESM.docx]

**Galectin-3 as a potential prognostic biomarker of severe COVID-19 in SARS-CoV-2 infected patients**

Eduardo Cervantes-Alvarez^1, 5#^, Nathaly Limon-de la Rosa^1#^, Moises Salgado-de la Mora^2^, Paola Valdez-Sandoval^2^, Mildred Palacios-Jimenez ^1,6^, Fatima Rodriguez-Alvarez^1,6^, Brenda I. Vera-Maldonado^1,6^, Eduardo Aguirre-Aguilar^2^, Juan Manuel Escobar-Valderrama^2^, Jorge Alanis-Mendizabal^2^, Osvely Méndez-Guerrero^1^, Farid Tejeda-Dominguez^4^, Jiram Torres‑Ruíz^3^, Diana Gómez‑Martín^3^, Kathryn L Colborn^7^, David Kershenobich^1^, Christene A Huang^7*^, Nalu Navarro-Alvarez^1,4,7*^

**Supplementary fig. S1: Flowchart illustrating patient selection for the study**

**Supplementary fig. S2: Smoothing splines of galectin-3, CRP and albumin. a,** Galectin-3 showed a non-linear relationship with the patients’ outcome (severe=1 and non-severe=0), for which it was used as a binary variable in the logistic regression analysis. **b,c,** Smoothing splines of CRP (**b**) and albumin (**c**), showed a linear relationship with outcome, thus both were used as continuous variables in further analysis. Data in **a,b,c** were computed with 4 knots.
